# Draft Genome Sequence of Citrobacter freundii AYS58, a Potential Plant Growth-Promoting Endophyte

**DOI:** 10.1128/mra.00142-23

**Published:** 2023-04-27

**Authors:** Ayomide Emmanuel Fadiji, Ayansina Segun Ayangbenro, Olubukola Oluranti Babalola

**Affiliations:** a Food Security and Safety Focus Area, Faculty of Natural and Agricultural Sciences, North-West University, Mmabatho, South Africa; Wellesley College

## Abstract

Here, we report the draft genome sequence of Citrobacter freundii AYS58, an endophyte isolated from the roots of a maize plant in Mafikeng, South Africa. The genome was 5,569,547 bp and exhibited a GC content of 50.5% and 5,904 genes, with 5,658 coding sequences, 3 rRNAs, 82 tRNAs, and 1 CRISPR.

## ANNOUNCEMENT

Citrobacter freundii is a Gram-negative and facultative anaerobic bacterium that belongs to the family *Enterobacteriaceae*. C. freundii, one of the most common species associated with plants, possesses multiple plant growth-promoting (multi-PGP) traits and can be used as a biofertilizer in agriculture (1). Several studies have reported the endophytic occurrence of C. freundii in various crops, such as rubber (1) and rice (2). C. freundii AYS58, a nonrhizobial endophyte, was isolated from the roots of Zea mays (maize) from Mafikeng, South Africa (25°47′25.24056″S, 25°37′8.17464″E). Healthy maize plants were selected, and their roots were detached and surface sterilized (3–5). The uncrushed sterilized roots were placed on tryptone soya agar (TSA) plates as a control to check the efficacy of surface sterilization. One gram of plant material was weighed and macerated. The macerated samples were plated on TSA and incubated at 28 ± 2°C for 3 days. A single colony was selected and confirmed as C. freundii through morphological and biochemical tests. The isolate was observed to possess plant growth-promoting traits, such as the solubilization of phosphate (6) and the production of ammonia and siderophores (7), as well as indole-3-acetic acid (8). The presence of these notable plant growth-promoting traits mediated the sequencing of the genome, which further confirmed this strain as C. freundii.

Genomic DNA was extracted from the pure culture on solid agar using the Quick-DNA extraction kit (Zymo Research, USA). The genome of strain AYS58 was sequenced at Novogene Company Limited, Singapore. A paired-end (PE) sequencing library was prepared from the DNA sample using the Illumina Nextera DNA Flex library preparation kit. The PE Illumina library was loaded into the NovaSeq 6000 instrument (2 × 150 bp) for cluster generation and sequencing. Whole-genome sequencing was visualized using KBase (9), which yielded 13,552,972 reads, representing a genome coverage of 365×. The read quality was examined using FastQC version 1.0.4 (10). Trimmomatic version 1.2.14 was used to filter out the adapter regions and low-quality reads from the data (11). After the filtering and trimming steps, a total of 13,280,536 reads were obtained. The genome assembly was created using SPAdes version 3.15.3 (12). Default parameters were used for all software unless otherwise specified. A total assembly size of 5,569,547 bp was obtained, with 766 contigs, 50.5% GC content, an *N*_50_ value of 441,722 bp, and an *L*_50_ value of 4. This file was then used to perform a BLAST assessment against GTDB-Tk version 1.7.0 (13).

The prediction of gene functions was carried out using the NCBI Prokaryotic Genome Annotation Pipeline (PGAP) (14). The results revealed a total of 5,904 genes, including 5,658 coding DNA sequences (CDSs), 129 Pseudogenes, 129 noncoding sequences, 3 rRNAs, 82 tRNAs, and 17 noncoding RNAs [ncRNAs]).

### Data availability.

The whole-genome sequence of Citrobacter freundii strain AYS58 has been deposited at DDBJ/ENA/GenBank under the accession number JAOEIR000000000. The version described in this paper is version JAOEIR000000000. The raw reads are available under the BioProject accession number PRJNA882746 and the BioSample accession number SAMN30948446. The sequence data obtained in this work have been deposited in the NCBI Sequence Read Archive under the accession number SRR21656364.
